# Interactions between fecal bacteria, bile acids and components of tomato pomace

**DOI:** 10.1007/s10068-018-0527-6

**Published:** 2018-12-08

**Authors:** Krzysztof Dziedzic, Danuta Górecka, Artur Szwengiel, Jan Michniewicz, Agnieszka Drożdżyńska, Jarosław Walkowiak

**Affiliations:** 1 Institute of Food Technology and Plant Origin, Poznan University of Life Sciences, Wojska Polskiego 31, 60-624 Poznan, Poland; 2 Department of Gastroenterology and Metabolic Diseases, Poznan University of Medical Sciences, Szpitalna 27/33, 60-572 Poznan, Poland; 3 Department of Gastronomy Science and Functional Food, Poznan University of Life Sciences, Wojska Polskiego 31, 60-624 Poznan, Poland; 4 Department of Biotechnology and Food Microbiology, Poznan University of Life Sciences, Wojska Polskiego 48, 60-627 Poznan, Poland

**Keywords:** Cholic acid, Deoxycholic acid, In vitro digestion, Lithocholic acid, Tomato waste

## Abstract

The tomato pomace obtained during processing as a residue of tomato processing from large industry. The interactions between tomato pomace and fecal bacteria, bile acids during in vitro digestion were studied. Digestion was carried out by using bioreactor in anaerobic conditions. Tomato pomace can significantly affect the count of fecal bacteria and the solubility of bile acids in in vitro digestion due to bonding ability of their proteins/peptides. The availability and use of bile acids does not only depend on the interactions between bile acids and bacteria, but also the interactions of bile acids with digested food components. Tomato pomace characterized high dietary fiber content and its fractions: 17.64–21.53% for cellulose and 13.48–18.63% for lignin. Given our results we supposed that fecal bacteria can use primary bile acids, as their source of energy in an environment where carbon availability is limited.

## Introduction

Tomato pomace (TP) is the most abundant waste in fruit and vegetable industries (11 million tone of tomato waste including 4 million tone of tomato pomace). The chemical composition of TP varies depending on the agricultural procedures applied and technical processing (Bajerska et al., 2015). This is a rich source of some nutritive compounds, such as: protein and lysine (15–24%), fat (5–20%, mainly linoleic acid), total sugar (28–51%) and mineral substances (3–6%)—all figures given in dry basis. They can be an excellent source of bioactive substances used as additives in food production (Güneşer et al., 2015; Stinco et al., 2016). TP is a good source of dietary fibre (mainly cellulose, hemicellulose, and lignin) (Borguini and Ferraz Da Silva Torres, 2009). The waste products of food production still contain very large amounts of secondary metabolites—primarily polyphenolic substances likes flavanones (naringenin, glycosilated derivatives), flavonols (quercetin, rutin, and kaempferol) and carotenoids, such as lycopene (Bajerska et al., 2015; Böhm, 2012; Chen et al., 2013; Kun et al., 2006; Stinco et al., 2016). There have been several attempts to enrich food products by using pomace as a source of dietary fibre and other bioactive compounds. Dry tomato peel was used during production of meat products (Previtera et al., 2016), snacks (Dehghan-Shoar et al., 2010; Dehghan-Shoar et al., 2011), rye bread (Bajerska et al., 2015), and tomato pasta (Reboul et al., 2005). The physiological activity of food components such as dietary fibre, protein and phytosterols in reducing plasma cholesterol has been extensively reported. Various freeze dried tomato products such as powder, pomace, juice, and other products have been reported to lower plasma cholesterol in hamsters and humans. Several mechanism of lowering LDL (low density lipoprotein) fraction cholesterol level have been documented. Changes in the levels of plasma cholesterol, fecal cholesterol and intestinal cholesterol absorption have been discussed (Shao et al., 2013). TP can also be an excellent source for the production of some prebiotics because of its nutritive value for microbial growth (Del Valle et al., 2006). In literature, there is a lack of evidence of the role of food products enriched with dietary fibre fractions obtained from tomato pomace in stimulation or retardation of fecal bacteria growth, and the ability of fecal bacteria in the management of bile salts and bile acids (Dziedzic et al., 2016). Begley et al. (2005) described that *E. coli* is considered to be very bile resistant and can be isolate from gallbladder and bile of humans. *E. coli* growth was observed in duodenum of a gastrointestinal model in the presence of very high concentration of bile, while Gram-positive bacteria in the same environment were deactivated. They showed also that bile tolerance is strain-specific. The effect of bile tolerance on a collection of 38 *Lactobacillus* strains were described: 5 strains were tolerant to bile of most than 0.3% concentration, whilst the growth of other strains was delayed. Among *Bifidobacterium* strains, *B. infantis* had the best survival rate but *B. longum* had the lowest (Begley et al., 2005).

Novelty of this experiment is investigation of few factors, which can be responsible for lipids manage in in vitro model. Therefore the aim of this study was to evaluate the role of TP in the conditioning of fecal bacteria and their ability to bind primary and secondary bile acids.

## Materials and methods

### Materials

TP used for this research was obtained from a Polish Food Industry Company “HJH Polska Sp. z. o. o.” (*Grandimat* variety, ripe and ready for consumption, from harvests in 2013 (TP1) and 2014 (TP2). TP was dried using a lyophilizer, and then kept at room temperature in the dark. Before the analysis, the sample was ground in a Foss Tecator mill (Hillerod, Sweden). Following reagents were used: pepsin, pancreatin, bile acids (cholic, deoxycholic and lithocholic acid), sodium bicarbonate, acetic acid, propionic acid, lactic acid, butyric acid purchased from Sigma-Aldrich (Seelze, Germany); hydrochloric acid, sodium hydroxide, ethanol, acetone, neutral disodium versenate dehydrate, disodium tetraborate decahydrate, disodium hydrogen phosphate, ethylene glycol, sulfuric acid (Poch, Gliwice, Poland); *N*-cetyl-*N*,*N*,*N*-trimethylammoniumbromid, Kanamycine Esculine Azide Agar, TOS (transoligosacharide) propionate agar medium with MUP (Lithium Mupirocin), Endo agar, and MRS agar. (Merck, Darmstadt, Germany); thermostable α-amylase (Novozymes, Bagsvaerd, Denmark).

### In vitro digestion

The digestive process (30 g of sample) was carried out according to Dziedzic et al. (2015; 2016) The size of the reaction tank was modified (1 L). The environment of the stomach, small and large intestine was reproduced as closely as possible (Ulleberg et al., 2011) with its pH and enzymes i.e. pepsin (0.576 g in 12 mL of 0.1 M sterilized hydrochloric acid)—the first stage of digestion, pancreatin (0.12 g), and bile acids (cholic acid, CA; deoxycholic acid, DCA; lithocholic acid, LHA, each 0.36 g)—mixed together in 30 mL of 0.1 M sterilized sodium bicarbonate as a suspension—the second stage of digestion. Subsequently, a mix of fecal bacteria, previously isolated from a healthy 24 year old male, was added at stage 2 (after 30 min., anaerobic conditions), in the amount of 10^4^–10^6^ CFU/mL. Next after 2 h the pH was changed again for 8.0—the third stage of digestion (18 h, large intestine). A bioreactor (300 mL of total volume, Sartorius Stedim, Biostat B Plus, Goettingen, Germany) was used as the in vitro digestive tract, and samples were obtained from three stages of digestion (1—duodenum, pH 6.0; 2—ileum, pH 7.2; 3—colon, pH 8.0). The simulation of the gastrointestinal tract was conducted at 37 °C, in anaerobic conditions and at the stirring speed of 200 rpm.

### Dietary fiber assay

#### Total dietary fiber (TDF)

The content of TDF, soluble dietary fiber (SDF) and insoluble dietary fiber (IDF) was analyzed using the enzymatic method (Dziedzic et al., 2016). The following enzymes were used: thermostable α-amylase (Termamyl 120 L, pH 6.0, 90 °C, 15 min.); pepsin (pH 1.5, 40 °C, 1 h), and pancreatin (pH 6.8, 40 °C, 1 h). Analyses were performed using a Fibertec System 1023 apparatus (Foss, Hillerod, Sweden).

#### Detergent fiber determination

The content of neutral dietary fiber (NDF), consisting of acid detergent fiber (ADF) and acid detergent lignin (L), was determined using the detergent method, previously used by Dziedzic et al. (2016). Thermostable α-amylase was used to digest starch. The reagents applied to estimate the content of neutral detergent fiber (NDF) were: neutral disodium versenate dehydrate, disodium tetraborate decahydrate, disodium hydrogen phosphate, ethylene glycol and redistilled water. The reagents used to estimate the content of ADF were: sulfuric acid (1 N, Poch, Gliwice, Poland, pure p.a.) and *N*-cetyl-*N*,*N*,*N*-trimethylammoniumbromid. The reagent used to estimate the content of L was sulfuric acid (72%). Hemicellulose (H) content was calculated from the difference between NDF and ADF. Cellulose (C) content was calculated from the difference between ADF and L. Analyses were conducted using a Fibertec System M 1020 apparatus by Tecator (Foss, Hillerod, Sweden).

### Bile acid assay

Bile acids (CA, DCA and LHA) were analyzed using LC–MS method described by Dziedzic et al. (2015; 2016). Ultra high-performance liquid chromatography electrospray ionization mass spectrometry analysis was performed using a DionexUltiMate 3000 UHPLC (Thermo Fisher scientific, Sunnyvale, CA, USA) coupled with a Bruker maXis impact ultrahigh resolution orthogonal quadrupole-time-of-light accelerator (qTOF) equipped with an ESI source and operated in the positive-ion Dean distance measure.

### Fecal bacteria identification (CFU)

Microbiological research was carried out according to international standards (Wohlsen et al., 2006). The count of *Enterococcus* spp., *Bifidobacterium* spp., *E. coli* and *Lactobacillus* spp. in the experimental samples were determined using the general pour plate technique on Kanamycine Esculine Azide Agar for *Enterococcus* spp., TOS agar with MUP Selective Supplement for *Bifidobacterium* spp., Endo agar for *E. coli*, and MRS agar for *Lactobacillus* spp.

### Short chain fatty acid assay (SCFA)

Determination of organic acids (acetic acid, propionic acid, lactic acid, butyric acid) was carried out using a UHPLC (VWR-HITACHI LaChrom Elite) system consisting of an autosampler (model L-2200), pump (model L-2130) and a UV detector (*L*-*2400*) connected in a series (Primec et al., 2017). Analyses were performed isocratically at a flow rate of 0.6 mL/min at 40 °C, on Rezex ROA—Organic Acid H+, 300 × 7.8 mm (Phenomenex) column. Standards (lactic acid—1.1, 0.55, 0.275, 0.11 g/L; acetic acid—1.0, 0.5, 0.25, 0.1 g/L; propionic acid—1.0, 0.5, 0.25, 0.1 g/L, and butyric acid- 0.55, 0.275, 0.1375, 0.055 g/L) were used.

### Statistical analysis

The experiments were executed in three independent trials. *T* test and Hierarchical cluster analysis were carried out. Tree plots were scaled to a standardized scale (dlink/dmax * 100). Non-hierarchical cluster analysis (k-means clustering) was performed to form a grouping of control/tomato pomace (TP1 and TP2) samples at the three stages of digestion (pH: 6.0, 7.2 and 8.0). Principal component analysis (PCA) technique was used to reduce the dimensionality of data and to present the samples in a new coordinate system. Statistica software, Version 10, StatSoft Inc. (Tulsa, OK, USA) was used to carry out statistical analysis.

## Results and discussion

Fat, protein, ash content, dietary fiber, and its soluble and insoluble fractions were estimated. Results of t-test for independent samples show significant differences between analyzed samples. The differences between TP1 and TP2 showed that year of harvest had influence for content of investigated substances in the samples, Table 1. TP1 had the highest content of fat, NDF, H and L in comparison to TP2.

**Table 1:** Characteristics of the tomato pomaces obtained in two consecutive years (TP1 and TP2), SD—the standard deviation (g/100 g of product)

Component	TP1* ± SD	TP2* ± SD
Fat	9.72^b^ ± 0.50	7.23^a^ ± 0.12
Protein	18.18^a^ ± 0.13	18.93^b^ ± 0.21
Ash	4.01^a^ ± 0.02	4.14^b^ ± 0.05
NDF	49.33^a^ ± 0.34	45.97^b^ ± 0.39
Cellulose	17.64^a^ ± 0.12	21.53^b^ ± 0.56
Hemicellulose	13.01^b^ ± 0.87	10.96^a^ ± 0.69
Lignin	18.69^b^ ± 0.67	13.48^a^ ± 0.18
SDF	0.61^a^ ± 0.08	0.87^b^ ± 0.06
IDF	45.27^a^ ± 0.56	49.16^b^ ± 0.57
TDF	45.88^a^ ± 0.62	50.03^b^ ± 0.63

IDF, insoluble dietary fiber; NDF, neutral detergent fiber; SDF, soluble dietary fiber; TDF, total dietary fiber

*Superscripts indicate significant difference in rows between means (*p* < 0.05)

**Fig. 1:**
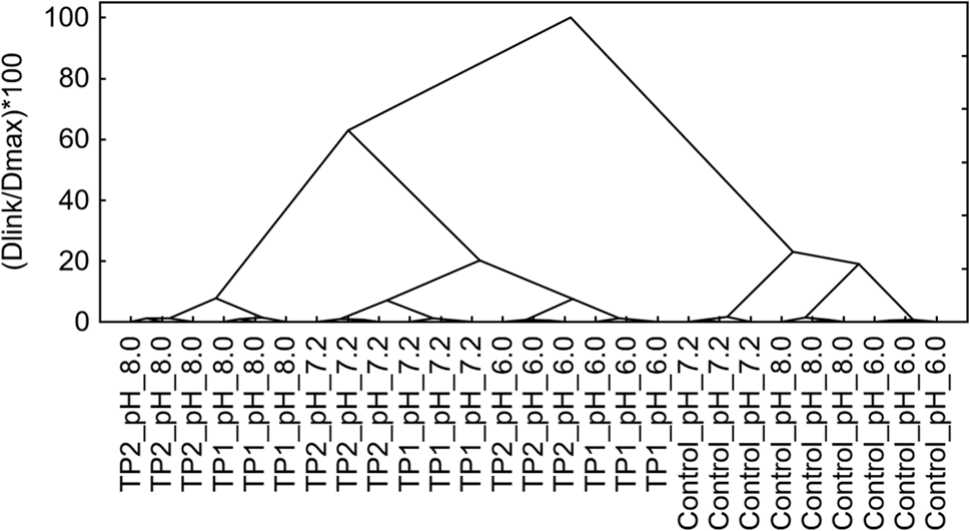
Results of the cluster analysis showing the relation between tomato pomace (TP1 and TP2) parameters and variation of number of viable bacteria, concentration of short-chain fatty acids and concentration of bile acids at the three stages of digestion (pH: 6.0, 7.2 and 8.0). The normalisation of scale tree to dlink/dmax * 100 was performed (d, distance; l, linkage; max, maximum of linkage Euclidean distance). Amalgamation rule: Ward’s method, distance metric: Euclidean distances

The in vitro digestion was carried out in three stages, where pH was 6.0, 7.2, and 8.0, respectively. The control sample consisted of a mixture of reagents and bacteria without TP. During in vitro digestion the concentration of individual bile salts, SCFA, and CFU were determined at each stage, Table 2. First, the exploration of two-dimensional data matrix using cluster analysis was conducted (Tables 1, 2, Fig. 1).

**Table 2:** Number of viable bacteria (CFU), concentration of short-chain fatty acids (SCFA) and concentration of bile acids at the three stages of tomato pomace digestion (pH: 6.0, 7.2 and 8.0)

Stage of digestion	pH_6.0	pH_6.0	pH_6.0	pH_7.2	pH_7.2	pH_7.2	pH_8.0	pH_8.0	pH_8.0
Sample	Control	TP1	TP2	Control	TP1	TP2	Control	TP1	TP2
*E. coli* (log CFU)	6.26 ± 0.23	6.39 ± 0.16	6.11 ± 0.09	6.78 ± 0.69	5.64 ± 0.11	5.86 ± 0.06	10.39 ± 0.37	6.84 ± 0.19	7.03 ± 0.11
*Enterococcus* (log CFU)	6.86 ± 0.18	6.70 ± 0.13	6.56 ± 0.08	6.90 ± 0.21	6.21 ± 0.19	6.55 ± 0.08	6.49 ± 0.17	9.98 ± 0.18	10.07 ± 0.23
*Lactobacillus* (log CFU)	6.88 ± 0.15	6.53 ± 0.11	6.90 ± 0.03	7.04 ± 0.11	6.27 ± 0.17	6.70 ± 0.16	8.58 ± 0.57	9.87 ± 0.48	10.00 ± 0.49
*Bifidobacterium* (log CFU)	3.46 ± 0.21	3.48 ± 0.1	3.60 ± 0.01	3.20 ± 0.08	3.41 ± 0.09	3.51 ± 0.14	0.00 ± 0.00	0.00 ± 0.00	0.00 ± 0.00
Lactic acid (g/L)	0.00 ± 0.00	0.97 ± 0.13	0.91 ± 0.05	0.26 ± 0.02	0.73 ± 0.03	1.09 ± 0.07	1.39 ± 0.08	5.33 ± 0.02	5.59 ± 0.00
Acetic acid (g/L)	0.02 ± 0.02	0.35 ± 0.03	0.52 ± 0.02	0.13 ± 0.01	0.63 ± 0.02	0.65 ± 0.04	0.24 ± 0.01	1.64 ± 0.00	1.58 ± 0.00
Propionic acid (g/L)	0.05 ± 0.02	0.64 ± 0.07	1.29 ± 0.06	0.48 ± 0.02	1.42 ± 0.02	2.05 ± 0.13	0.41 ± 0.03	1.56 ± 0.00	1.99 ± 0.00
Butyric acid (g/L)	0.00 ± 0.00	0.00 ± 0.00	0.00 ± 0.00	0.00 ± 0.00	0.00 ± 0.00	0.00 ± 0.00	0.04 ± 0.00	0.20 ± 0.01	0.24 ± 0.02
CA (mg/mL)	0.00 ± 0.00	18.20 ± 0.58	22.23 ± 0.47	28.83 ± 1.06	28.11 ± 0.51	25.76 ± 0.25	1.09 ± 0.01	22.02 ± 0.57	22.17 ± 0.39
DCA (mg/mL)	0.00 ± 0.00	3.47 ± 0.03	0.87 ± 0.03	23.66 ± 0.56	23.34 ± 0.18	22.27 ± 0.39	0.53 ± 0.00	18.38 ± 0.08	18.01 ± 0.02
LHA (mg/mL)	0.00 ± 0.00	0.49 ± 0.00	0.15 ± 0.00	5.02 ± 0.15	3.54 ± 0.01	3.95 ± 0.04	0.41 ± 0.01	9.14 ± 0.06	5.51 ± 0.06

The analysis of hierarchical tree showed 2 different groups of results (the height of the cut-off point 80%). The first cluster consists of control samples of high homogeneity, while the second cluster contains TP samples. We can conclude that TP samples had an effected on the fecal bacteria and the profile of analyzed substances during in vitro digestion (Table 2). It is possible to distinguish three subgroups of six elements each, which are specific to further stages of in vitro digestion (the height of the cut-off point 6%). However, the most diversified group consisted of samples obtained from the third stage of digestion (pH 8.0). At the second stage, k-means clustering was conducted, assuming a priori the number of groups equal to 3 based on the results of cluster analysis. The results of k-means analysis confirm the earlier observations as to the assignment of the samples to three groups. The evaluation of discriminant power of variables (*p* < 0.05) by using ANOVA method proves that only the concentration of DCA does not differ significantly within the analyzed groups (Table 3).

**Table 3:** K-means clustering, ANOVA results, evaluation of discriminant power of variables (*p* < 0.05); the final classification of control/tomato pomace (TP1 and TP2) samples at the three stages of digestion (pH: 6.0, 7.2 and 8.0)

Variable	Between SS*	df*	Within SS*	df	F*	*p* value*	Case	Distance*	Final classification
*E. coli*	8.8313	2	17.1687	24	6.170	0.0069	Control_pH_6.0	0.448	1
*Enterococcus*	25.4231	2	0.5769	24	528.860	0.0000	Control_pH_7.2	0.616	1
*Lactobacillus*	22.1700	2	3.8300	24	69.460	0.0000	Control_pH_8.0	0.608	1
*Bifidobacterium*	17.8108	2	8.1892	24	26.100	0.0000	TP1_pH_6.0	0.394	2
Lactic acid	25.1193	2	0.8807	24	342.280	0.0000	TP1_pH_6.0	0.366	2
Acetic acid	25.2065	2	0.7935	24	381.210	0.0000	TP1_pH_6.0	0.380	2
Propionic acid	18.5188	2	7.4813	24	29.700	0.0000	TP2_pH_6.0	0.318	2
Butyric acid	25.3556	2	0.6444	24	472.140	0.0000	TP2_pH_6.0	0.322	2
CA	9.6018	2	16.3982	24	7.030	0.0040	TP2_pH_6.0	0.330	2
DCA	3.4941	2	22.5060	24	1.860	0.1770	TP1_pH_7.2	0.337	2
LHA	14.8327	2	11.1673	24	15.940	0.0000	TP1_pH_7.2	0.338	2
Fat	24.3406	2	1.6594	24	176.020	0.0000	TP1_pH_7.2	0.309	2
Protein	25.9633	2	0.0368	24	8478.050	0.0000	TP2_pH_7.2	0.345	2
Ash	25.9760	2	0.0240	24	12,964.190	0.0000	TP2_pH_7.2	0.394	2
NDF	25.9003	2	0.0997	24	3118.380	0.0000	TP2_pH_7.2	0.373	2
Cellulose	25.2290	2	0.7710	24	392.650	0.0000	TP1_pH_8.0	0.243	3
Hemicellulose	25.2282	2	0.7718	24	392.240	0.0000	TP1_pH_8.0	0.216	3
Lignin	24.0645	2	1.9355	24	149.200	0.0000	TP1_pH_8.0	0.220	3
SDF	23.4196	2	2.5804	24	108.910	0.0000	TP2_pH_8.0	0.224	3
IDF	25.8610	2	0.1390	24	2233.130	0.0000	TP2_pH_8.0	0.229	3
TDF	25.8465	2	0.1536	24	2019.970	0.0000	TP2_pH_8.0	0.226	3

SS*, sum of squares between/within groups; df, degrees of freedom; F, statistical F-test; *p* value, probability value; distance, distance from respective cluster center; CA, cholic acid; DCA, deoxycholic acid; LHA, lithocholic acid; IDF, insoluble dietary fiber; NDF, neutral detergent fiber; SDF, soluble dietary fiber; TDF, total dietary fiber

**Fig. 2:**
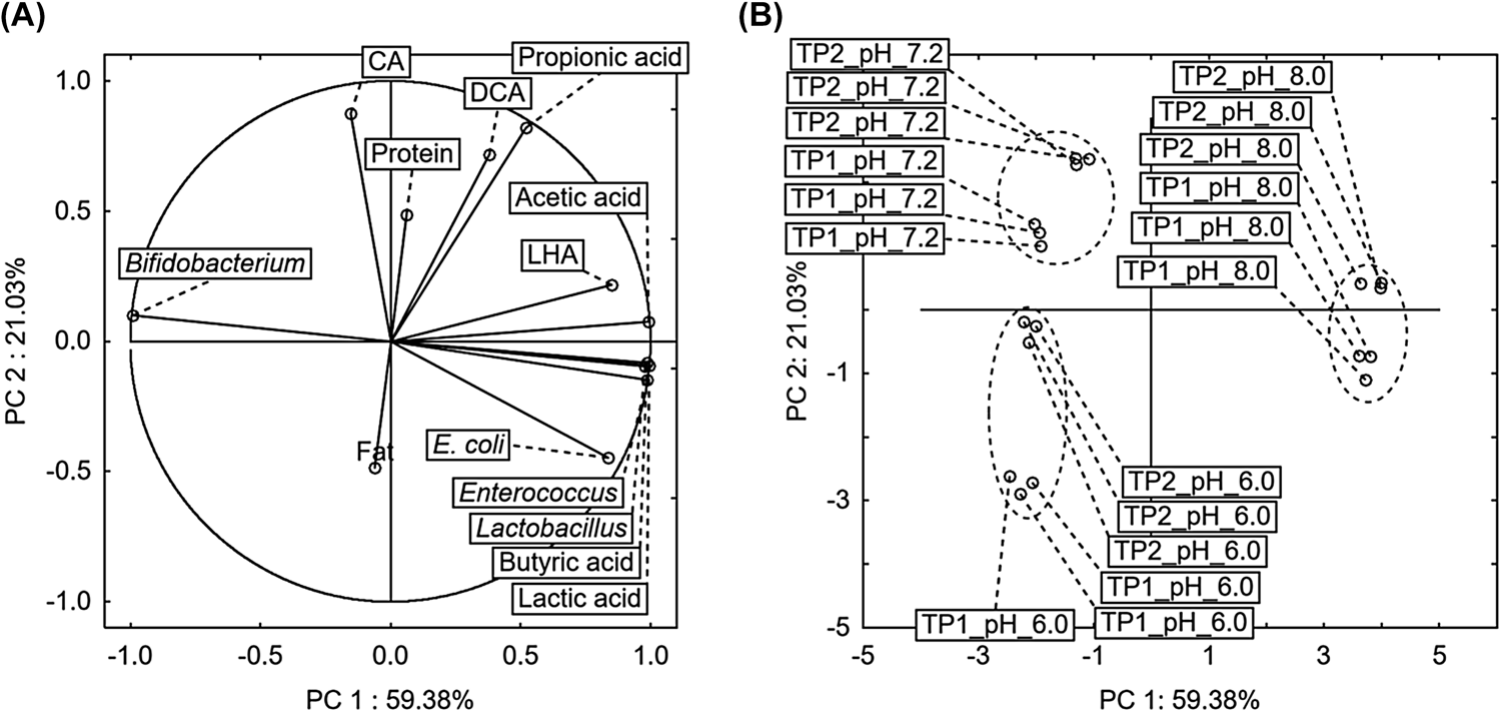
Tomato pomace samples (TP1 and TP2) at the three stages of digestion (pH: 6.0, 7.2 and 8.0) were presented in plot representing the PCA of loadings plot (**A**) and score plot (**B**); principal components—PC 1 and PC 2; CA, cholic acid; DCA, deoxycholic acid; LHA, lithocholic acid

To determine the relationships between variables, variables—samples, and samples alone, a PCA analysis was performed. The results were presented in Fig. 2 in a system of two principal components (PCs), explaining 80.41% of total variance. In this way the number of original dimensions was reduced from 13 original variables to 2 newly created (PC 1 and PC 2). Instances excluding control samples were examined, because they exhibited significant differences to TP samples, as demonstrated above. Solubility of bile acids in control samples was lower than in the case of TP samples, what is related to absence of short chain fatty acids (Table 2). A positive correlation was found between the concentration of LHA, acetic acid, lactic acid, butyric acid, and the count of *E. coli*, *Enterococcus* spp., *Lactobacillus* spp. (Fig. 2A), while a negative correlation was found for *Bifidobacterium* spp.

This indicates low survival rate of *Bifidobacterium* spp. in experimental environment. The concentration of LHA was highest at pH 8.0. Bile acids appear to be a major regulator of the gut microbiota. Other authors demonstrated toxic activity of secondary bile acids on some lactic acid bacteria in previous research (Dziedzic et al., 2016; Merritt and Donaldson, 2009). Bile acids have both direct effect on fecal bacteria and indirect effect through induced antimicrobial peptides (Ridlon et al., 2014). Some authors described DCA as a much more antimicrobial agent compared to CA (Begley et al., 2005). Saito et al. documented that *Enterococcus faecalis* is able to use carbon source from fatty acids (Saito et al., 2014), therefore we suppose that fecal bacteria can utilize LHA, and its concentration is lowered in control sample in the absence of other bioavailable carbon source. On the other hand in TP samples the concentration of secondary bile acids increases, which can be linked to the role of some fecal bacteria in biotransformation of primary bile acids into secondary bile acids (Dziedzic et al., 2015). Comprehensive vector bundle consists of protein, propionic acid, DCA and CA variables. Analyzing the concentration of DCA and CA in control samples (Table 2) it can be concluded that these samples are featured by low solubility in acidic pH (6.2), and good solubility in basic environment (pH 7.2), where their concentration increased (Dziedzic et al., 2016). Higher solubility of these acids in control samples affected their bioavailability (the only carbon component) for fecal bacteria. As a result, at the final stage of digestion (pH 8.0) a low concentration of these acids was observed (Table 2). Begley et al. (2005) described that *E. coli* growth was observed in the small intestinal compartments of digestive tract model in the presence of high concentrations of bile extract, whereas the content of Gram-positive bacteria decreased. Many authors suggested that Gram-positive bacteria are strain-specific taking into consideration tolerance to bile salts and their concentration (Chateau et al., 1994; Jacobsen et al., 1999; Zárate et al., 2000). Some evidence has shown that bile acids can be metabolized by some gut bacteria (Ruiz et al., 2013). The high concentration of these acids in TP samples does not only depend on the pH of the environment at individual stages of digestion, as confirmed by the location of vectors in Fig. 2A compared with samples in Fig. 2B. Proteins introduced together with pomace probably bond CA and DCA. These acids are negatively correlated with *E. coli* count, while remaining neutral to other analyzed bacteria. They also demonstrate positive correlation with propionic acid, which suggests a connection between the concentration of CA, DCA, and metabolites created by the bacteria.

It was found that fecal bacteria can use bile acids, i.e. CA and DCA, as their source of energy in an environment where carbon availability is limited. Furthermore, it was demonstrated that tomato pomace can significantly affect the count of fecal bacteria and the solubility of bile acids (CA and DCA) in in vitro digestion due to bonding ability of their proteins/peptides. Obtained results prove that the availability and use of bile acids does not only depend on the interactions between bile acids and bacteria, but also the interactions of bile acids with other food components. The analysis of the influence of bacteria on bile acid concentrations in models without the food component does not reflect real life scenarios. An addition of tomato pomace to food can improve the condition of human digestive tract. However, the mechanism of bile acid sorption by tomato pomace, especially proteins and dietary fiber, and its resorption in digestive tract remains to be clarified. This would allow to conclude which of the considered factors have a significant effect on bile acid sorption.
